# Workplace lactation support attenuates the association between early maternal return to work and preschool overweight/obesity in China

**DOI:** 10.3389/fpubh.2026.1881827

**Published:** 2026-09-09

**Authors:** Huan Liu

**Affiliations:** Nursing Department, Pingxiang Maternal and Child Health Hospital, Pingxiang, Jiangxi, China

**Keywords:** breastfeeding, childhood obesity, China, maternal employment, maternity leave, public health policy, workplace lactation support

## Abstract

**Background:**

Maternal return to paid work may disrupt infant feeding continuity, yet its relationship with childhood adiposity remains uncertain. This study examined associations between return-to-work timing, breastfeeding, workplace lactation support, and preschool overweight/obesity in China.

**Methods:**

This single-center cross-sectional study enrolled 1,633 mother–child dyads in Jiangxi Province, China, between February 2023 and August 2024. Children aged 36–72 months underwent standardized anthropometric assessment, and mothers reported return-to-work timing, breastfeeding duration, formula introduction, complementary feeding, and workplace lactation support. Multivariable logistic regression estimated adjusted odds ratios (ORs), with exploratory stratified and sensitivity analyses assessing heterogeneity by residence, maternal education, and workplace support.

**Results:**

Overall, 458 children (28.0%) were overweight or obese. Among mothers, 733 (44.9%) did not return to paid work during the first postpartum year; 23 (1.4%) returned before 6 weeks, 260 (15.9%) at 6–15 weeks, and 617 (37.8%) at ≥16 weeks. Relative to no return, return at 6–15 weeks was associated with higher adjusted odds (OR 1.85, 95% CI 1.36–2.50), whereas return at ≥16 weeks was not (OR 1.06, 0.82–1.35). The estimate for return before 6 weeks was uninformative (*n* = 23; OR 2.23, 0.95–5.22), and the trend test was non-significant (*p* = 0.30). Inverse associations were observed for exclusive breastfeeding ≥6 months (OR 0.41, 0.26–0.63) and any breastfeeding 6–11 months (OR 0.61, 0.42–0.88); all pre-specified feeding–work interaction terms were null. In exploratory stratified analyses, return before 16 weeks was associated with overweight/obesity among mothers with low workplace support (OR 3.84, 2.25–6.56) but not high support (OR 1.08, 0.72–1.61; *p*-for interaction<0.001).

**Conclusion:**

Return at 6–15 postpartum weeks was the only timing category robustly associated with preschool overweight/obesity; no monotonic dose–response was evident. Sustained breastfeeding was associated with lower odds. The heterogeneity by workplace lactation support is exploratory in a study not powered for interaction and requires confirmation.

## 1 Introduction

Childhood obesity represents a critical global health challenge. Among children aged 0–5 years, the prevalence of overweight and obesity increased from 32 million to 41 million between 1990 and 2016, with the steepest rise occurring in low- and middle-income countries (1–4). Early childhood adiposity strongly predicts metabolic syndrome, type 2 diabetes mellitus (T2DM), and cardiovascular disease, underscoring the urgency of early prevention (5). Because excess weight established in early childhood often tracks into later childhood and adult life, identifying modifiable exposures during infancy and the preschool years remains central to obesity prevention (6, 7).

The first 1,000 days represent a critical window during which metabolic programming influences long-term health trajectories (8, 9). Breastfeeding has been associated with lower odds of childhood overweight/obesity in many observational studies; however, the magnitude and dose–response pattern remain influenced by differences in breastfeeding definitions, confounding structures, and population context (10). Proposed mechanisms underlying these associations include bioactive hormones regulating satiety, lower protein content reducing insulinogenic responses, and human milk oligosaccharides promoting beneficial gut microbiota (11, 12). Despite World Health Organization (WHO) recommendations for 6 months of exclusive breastfeeding, global adherence remains inadequate (13), and breastfeeding cessation rates increase sharply when mothers re-enter paid employment (14, 15).

Expanding female labor force participation has intensified the structural conflict between employment demands and sustained lactation globally. Early return to work consistently curtails breastfeeding duration and accelerates formula introduction (15, 16); however, its effect on childhood obesity remains uncertain. Evidence from high-income countries shows inconsistent associations (17–20), and workplace lactation support as an effect modifier remains under-examined. This knowledge gap is critical in rapidly developing economies experiencing simultaneous increases in female employment and childhood obesity. China exemplifies this transition. Rapid economic development has driven a shift from undernutrition to over-nutrition within one generation, with childhood overweight/obesity prevalence reaching 10.4% in urban areas (19–23). Despite statutory 98-day maternity leave, surveys of Chinese working mothers report that job-related factors—short leave, inflexible scheduling, and limited employer accommodation—are consistently associated with earlier resumption of paid work and shorter breastfeeding duration (23, 24). Whether financial necessity is itself a direct determinant of early postpartum return in this setting has not been established empirically, and this study advances it here only as a plausible mechanism requiring dedicated work. National surveillance indicates that while breastfeeding initiation is high, exclusive breastfeeding rates at 6 months fall short of the 50% target, declining precipitously upon maternal workforce re-entry (25, 26). China presents unique obesogenic vulnerabilities. The cultural tradition of zuo yuezi (postpartum confinement) provides robust early support; however, it dissipates abruptly upon workforce re-entry (27, 28). Moreover, inadequate workplace lactation infrastructure—private rooms, protected breaks, and milk storage—creates barriers that often force premature formula use (28, 29).

Despite this context, two critical knowledge gaps persist. First, evidence from China remains limited regarding the combined association of postpartum return-to-work timing and breastfeeding duration with childhood adiposity. Second, comprehensive workplace lactation support has not been evaluated as an effect modifier in this pathway. Studies of workplace lactation provision examine breastfeeding continuation as the endpoint and do not extend to child weight outcomes. Consequently, no study has assessed return-to-work timing, breastfeeding duration, and workplace lactation provision together in a single population, and workplace provision has not been evaluated as a modifier of the association between employment timing and child adiposity. Evidence from China is particularly limited, despite the concurrent rise in female labor participation and childhood obesity (14, 30, 31).

The study hypothesized that (i) early postpartum return to work (<16 weeks) and short breastfeeding duration (<6 months) would be independently and potentially jointly associated with a higher prevalence of childhood overweight/obesity, and that (ii) workplace lactation support would modify this association, with the effect of early return being markedly attenuated in settings with adequate lactation support. Clarifying these associations may inform structural workplace and policy interventions that support maternal employment while promoting breastfeeding continuation and healthy child growth.

## 2 Methods

### 2.1 Study design and participants

This single-center observational study combined a cross-sectional survey of maternal employment and infant feeding practices with the retrospective extraction of prospectively recorded longitudinal growth data from electronic medical records and child health booklets conducted at the Pingxiang Maternal and Child Health Hospital, Jiangxi Province, China. This center provides integrated maternal–child services with systematic prospective documentation of serial anthropometry from birth onwards in standardized child health booklets maintained at every well-child visit. The cross-sectional survey component captured maternal exposures (employment timing, workplace support, and feeding practices) at a single study visit; the longitudinal growth component used retrospectively extracted, prospectively recorded weight, and length/height measurements from birth through the survey date. Mother–child dyads were consecutively recruited during routine well-child visits between February 2023 and August 2024.

The eligible participants included biological mothers aged ≥18 years with singleton live births who were primary caregivers at survey time. Children were required to be aged 36–72 months with complete birth anthropometrics and at least three serial growth measurements during the first 24 months to support early growth record indicators. Exclusion criteria included major congenital anomalies, chronic growth-affecting conditions (severe cardiac, renal, or endocrine disorders; chromosomal abnormalities), gestational age <32 weeks, birth weight <1,500 g, multiple gestations, and missing primary exposure or outcome data. The sample size calculation required 1,400 dyads to detect an OR of 1.30 for early (<16 weeks) versus late (≥16 weeks) return, with 80% power, *α* = 0.05, and 28% baseline prevalence; anticipating 15% non-participation, the study targeted 1,650 dyads. This calculation addressed the main effect of return timing only. Detecting a multiplicative interaction of comparable magnitude conventionally requires approximately a fourfold larger sample, a requirement the study was not designed to meet, and interaction and stratified analyses are accordingly exploratory throughout. Among 1,847 eligible dyads, 1,633 (88.4%) had complete primary exposure and outcome data and formed the analytic sample. Individual-level data were not retained for those who declined or were excluded, as the protocol did not provide for collecting identifiable information from non-consenting families; participants and non-participants could not therefore be compared. Covariate missingness was limited and was addressed in pre-specified sensitivity analyses.

### 2.2 Data collection

#### 2.2.1 Maternal survey

Trained nurses administered structured 45 min computer-assisted interviews adapted from validated instruments, including the China Health and Nutrition Survey (32–36). The survey captured: (1) sociodemographic characteristics (age, education, marital status, household income, residence); (2) retrospectively recalled employment and workplace conditions specific to the first postpartum year (i.e., conditions that applied during the period before the child reached 12 months of age, not current employment status), including pre-pregnancy employment, exact week of postpartum return [defined as ≥10 h/week], maternity leave duration, and workplace breastfeeding support indicators—lactation room availability, permitted breaks, and milk storage as experienced during the postpartum employment period; and (5) infant feeding history using life-course calendars with memory anchors at 1, 3, 6, 9, and 12 months. Interviewers explicitly instructed mothers to recall their employment and workplace conditions as they existed during the child’s first year of life and prompted recall using the temporal anchors of the life-course calendar to minimize conflation with current employment characteristics.

The data collected included breastfeeding initiation timing, exclusive breastfeeding duration per WHO criteria (defined strictly as breast milk only with no formula, water, or other liquids permitted, except oral rehydration solution, vitamins, and prescribed medicines; interviewers administered standardized probing questions to confirm true exclusivity at each recalled time point) (37, 38), any breastfeeding duration, formula introduction timing, solid food introduction timing, and predominant feeding method during the first year. Mothers were encouraged to consult child health booklets to minimize recall bias. Single-item breastfeeding duration questions showed good agreement (*κ* = 0.79) with prospectively collected data within5 years (35, 36, 39–41).

#### 2.2.2 Medical record abstraction

Two trained assistants independently extracted perinatal and growth data from electronic medical records and child health booklets. The variables included maternal age, pre-pregnancy weight and height, parity, gestational complications (gestational diabetes per the International Association of Diabetes and Pregnancy Study Groups criteria; hypertensive disorders per the American College of Obstetricians and Gynecologists definitions) (42–45), gestational age, delivery mode, birth weight, birth length, infant sex, and serial weight and length/height measurements from birth through survey. Inter-rater agreement in a 10% subsample yielded intra-class correlation coefficients of 0.998 and 0.996 for birth weight and length, respectively.

#### 2.2.3 Anthropometric assessment

Trained nurses performed anthropometric measurements per WHO protocols (46, 47). Weight was measured to 0.1 kg using daily-calibrated electronic scales (SECA 813; Hamburg, Germany). Height was measured to 0.1 cm using wall-mounted stadiometers (SECA 213) with the child’s head in the Frankfurt horizontal plane; two measurements were averaged, with a third measurement if the discrepancy exceeded 0.5 cm. Body weight and height were the primary anthropometric measures used for outcome classification; all equipment underwent scheduled calibration verification at the start of each clinic session. All personnel demonstrated inter-rater reliability (ICC > 0.95).

### 2.3 Variable definitions

#### 2.3.1 Primary exposure

The primary exposure was return-to-work timing, defined as the number of completed postpartum weeks when mothers resumed regular paid work (≥10 h/week). Mothers not engaged in paid work during the first postpartum year were classified as “no return to paid work.” The primary analysis used four *a priori* categories: (1) no return to paid work (reference), (2) return <6 weeks, (3) return 6–15 weeks, and (4) return ≥16 weeks, to characterize dose–response across the postpartum employment continuum. The 6-week cutoff was selected *a priori* as a policy-relevant threshold approximating the early postpartum period during which lactation establishment and return-to-work transitions commonly overlap (48–50). Stratified and sensitivity analyses employed a dichotomous comparison of return <16 weeks versus ≥16 weeks, aligning with this threshold and improving statistical power in subgroup analyses.

Among employed mothers, a composite workplace support score (range 0–3) was summed from three binary indicators: lactation room availability, permitted breastfeeding breaks, and milk storage facilities. Each component represents a distinct, non-substitutable infrastructural prerequisite for sustained workplace lactation—spatial privacy for expression, protected time for milk removal to maintain supply, and refrigerated storage for expressed milk safety—consistent with WHO and International Labor Organization Convention 183 guidance, which treats each as an independent necessity. As these items constitute formative rather than reflective indicators of workplace lactation infrastructure, internal consistency statistics are not applicable; each facility is an independent necessity rather than an interchangeable manifestation of a single underlying latent construct. The three items were selected *a priori* to map onto the functional prerequisites for workplace expression specified in WHO guidance and ILO Maternity Protection Convention 183—private space, protected time, and safe storage—each treated in those instruments as independently necessary. No pre-existing validated instrument covering these domains was available for Chinese working mothers, so a purpose-built index was required. Scores were dichotomized into high support (≥2 facilities) and low support (≤1 facility). This threshold was fixed before outcome analysis and was not chosen on model fit: it coincides with the sample median and reflects the requirement that at least two prerequisites coincide for expression to be practicable, since private space without protected time, or protected time without private space, does not permit expression to occur. Three alternative operationalizations were pre-planned as sensitivity analyses: the ordinal score entered continuously, a stricter contrast of complete (score 3) against incomplete (score 0–2) provision, and stratification on each component separately.

#### 2.3.2 Breastfeeding and feeding practice variables

Exclusive breastfeeding duration was categorized as never/0 months, <3 months, 3–5 months, and ≥6 months. Any breastfeeding duration was categorized as never breastfed, <6 months, 6–11 months, and ≥12 months and analyzed continuously (months) and dichotomously (<6 vs. ≥6 months). Formula feeding patterns included early introduction before 6 months (yes/no) and introduction timing, operationalized as first month versus thereafter. Complementary feeding timing was categorized as <4 months, 4-5 months, and ≥6 months. To assess potential multicollinearity among feeding practice variables, variance inflation factors (VIFs) were computed for all predictors in the primary logistic regression model; no VIF exceeded 3.1 (range 1.1–3.1), below conventional thresholds of concern (VIF > 5). Sensitivity analyses entering exclusive breastfeeding duration and formula introduction timing in separate models produced adjusted ORs for return-to-work timing within 3% of those from the full model, confirming that multicollinearity did not meaningfully influence estimates. Breastfeeding variables were harmonized before analysis using a pre-specified logical hierarchy.

Breastfeeding initiation was treated as the master indicator for any breastfeeding; therefore, mothers who reported no breastfeeding initiation were classified as having never breastfed and assigned an any-breastfeeding duration of 0 months. Among mothers who initiated breastfeeding, duration categories were derived from the reported months of any breastfeeding. Mothers who were still breastfeeding at the time of the survey were classified as having an any-breastfeeding duration of ≥12 months.

#### 2.3.3 Outcomes

*Primary outcome*: Overweight/obesity, defined as BMI-for-age *z*-score ≥+1 per WHO 2006 Child Growth Standards (WHO Anthro version 3.2.2 for children <5 years; AnthroPlus version 1.0.4 for older children). BMI was calculated as weight (kg) divided by height squared (m^2^).

*Secondary outcomes*: BMI-for-age *z*-score was analyzed as a continuous variable to complement the primary binary classification. Age- and sex-standardized BMI, weight, and height *z*-scores were computed using WHO 2006 Child Growth Standards (WHO Anthro version 3.2.2 for children aged <5 years; AnthroPlus version 1.0.4 for older children) and examined descriptively across return-to-work timing and breastfeeding duration categories. Continuous BMI-for-age *z*-score was modeled as an outcome using multivariable linear regression with the same covariate structure as the primary logistic regression model, reported as a pre-specified sensitivity analysis to assess the robustness of associations to the choice of outcome operationalization. Individual weight status categories (underweight, normal weight, overweight, and obese) were additionally reported descriptively by breastfeeding duration and return-to-work timing to characterize the full distribution. Two serial growth indicators extracted from child health booklet records were examined descriptively: rapid weight gain (weight *z*-score increase >0.67 SD from birth to 24 months, corresponding to upward crossing of at least one major percentile band) and growth faltering (downward crossing of ≥2 major length/height percentile lines).

#### 2.3.4 Covariates

Covariates were selected *a priori* based on a literature review and directed acyclic graphs. Maternal covariates included age at delivery, education, marital status, residence, household income quintile, pre-pregnancy body mass index (BMI), and parity. Pregnancy and delivery variables included gestational diabetes, hypertensive disorders, gestational age, delivery mode, and neonatal complications. Child variables included sex, birth weight, birth length, and age at survey.

### 2.4 Statistical analysis

Baseline characteristics are summarized by return-to-work timing using median (interquartile range) for continuous variables, with mean (standard deviation) additionally reported for age- and sex-standardized *z*-scores to aid interpretation, and frequencies (percentages) for categorical variables. The distribution of every continuous variable was assessed before analysis using the Shapiro–Wilk test together with visual inspection of histograms and normal quantile–quantile plots. All continuous variables—maternal age at delivery, pre-pregnancy BMI, household income quintile, gestational age, birth weight, birth length, child age at survey, exclusive and any breastfeeding duration, maternity leave duration, and weight, height, and BMI z-scores—departed significantly from normality (all Shapiro–Wilk *p* < 0.05). Between-group comparisons of continuous variables therefore used the Kruskal–Wallis test throughout; analysis of variance was not used at any point. Categorical variables were compared using *χ*^2^ or Fisher’s exact tests. Missing data ranged from 0 to 6.8%, with complete data for 91.1% (*n* = 1,487), concentrated in workplace support (6.8%), complementary feeding timing (3.4%), and maternal education (2.1%). Little’s test (*χ*^2^ = 142.3, d*f* = 156, *p* = 0.31) indicated that the data were missing completely at random. Primary analyses used multiple imputation by chained equations (mice v3.16.0), generating 20 imputed datasets over 20 iterations (seed = 2023). Predictive mean matching was applied to continuous variables, logistic regression to binary variables, and polytomous regression to unordered categorical variables. The imputation model included every covariate from the analysis models, along with the outcome. Dyads missing the primary exposure or outcome were excluded beforehand, and neither was imputed. Trace plots assessed convergence, and estimates were pooled using Rubin’s rules. Complete-case analysis in the 1,487 dyads with complete covariate data served as a sensitivity analysis. Multivariable logistic regression assessed the associations between return-to-work timing and childhood overweight/obesity. Model 1 adjusted for child age and sex; Model 2 added maternal sociodemographic factors; and Model 3 (primary) added pregnancy complications, birth characteristics, and feeding practices. Linear trend was tested by treating exposure as ordinal. Effect modification was assessed by stratification according to residence (urban vs. rural), maternal education (≤upper secondary vs. ≥vocational/tertiary), and workplace support (high vs. low). Multiplicative interactions were tested using likelihood ratio tests for the following pre-specified terms: short breastfeeding × early return, long breastfeeding × low support, and early formula × employment.

The following sensitivity analyses were conducted to examine robustness: (1) excluding preterm births (<37 weeks), (2) excluding low birth weight (<2,500 g), (3) return-to-work timing entered as a continuous variable (weeks) to assess dose–response, and (4) continuous BMI-for-age *z*-score as the outcome in multivariable linear regression. As a supplementary analysis, models were re-estimated by restricting the sample to the 1,172 mothers employed before pregnancy (of whom 900 returned to paid work), with ≥16-week returners as the reference group, to address potential heterogeneity within the primary “no return to paid work” reference category. Three interaction terms were pre-specified before data analysis: short breastfeeding duration (<6 months) × early return to work (<16 weeks), long breastfeeding duration (≥6 months) × low workplace support, and early formula introduction × maternal return to work; these were tested using likelihood ratio tests within the multivariable logistic regression framework. Because the primary hypotheses were pre-specified, regression estimates are presented with nominal two-sided 95% CIs and *p*-values. Stratified and sensitivity analyses were considered exploratory and interpreted according to consistency, precision, and biological plausibility. Secondary stratified analyses by residence, education, and workplace support were exploratory and were not corrected for multiple comparisons; these subgroup results should be interpreted as hypothesis-generating rather than confirmatory. All tests were two-sided (*α* = 0.05). Analyses were performed using R version 4.3.1 (R Foundation for Statistical Computing, Vienna, Austria) with tidyverse (v2.0.0), mice (v3.16.0), FactoMineR (v2.8), and tableone (v0.13.2).

## 3 Results

### 3.1 Sample characteristics and return-to-work timing

The analytic sample included 1,633 mother–child dyads. Children’s median age at survey was 53.4 months (IQR 44.3–62.7). Among the mothers, 733 (44.9%) did not return to paid work during the first postpartum year, 23 (1.4%) returned before 6 weeks, 260 (15.9%) returned at 6–15 weeks, and 617 (37.8%) returned at ≥16 weeks; 283 mothers (17.3%) returned within the first 16 postpartum weeks (Table 1). Maternal sociodemographic and pregnancy characteristics were broadly comparable across the return-to-work groups (Table 1). Residence type differed significantly (*p* < 0.001): urban residence was more prevalent among mothers who returned at 6–15 weeks (70.0%) and at ≥16 weeks (71.6%) than among non-returners (62.8%) or mothers who returned before 6 weeks (43.5%). Maternal age, education, household income, pre-pregnancy BMI, parity, gestational complications, gestational age, mode of delivery, and birth anthropometrics were similar across groups (all *p* ≥ 0.10; Table 1). At survey, 290 children (17.8%) were overweight, and 168 (10.3%) were obese, yielding a combined overweight/obesity prevalence of 28.0% (*n* = 458). Child weight status differed significantly across return-to-work groups (*p* = 0.002). Combined overweight/obesity was higher among children of mothers returning before 6 weeks (43.5%; 10/23) and at 6–15 weeks (38.5%; 100/260) than among non-returners (25.2%; 185/733) and ≥16-week returners (26.4%; 163/617).

**Table 1:** Maternal and child baseline characteristics by maternal return-to-work timing.

Characteristic	Overall (*N* = 1,633)	No return (*n* = 733)	Return <6 wk (*n* = 23)	Return 6–15 wk (*n* = 260)	Return ≥16 wk (*n* = 617)	*p*-value
Maternal characteristics						
Age at delivery, years	28.9 (26.3–31.6)	28.9 (26.4–31.6)	27.5 (25.2–29.7)	29.2 (26.5–31.9)	28.7 (26.2–31.7)	0.29
Education level						0.53
Primary/middle school	367 (22.5)	169 (23.1)	3 (13.0)	65 (25.0)	130 (21.1)	
High school	253 (15.5)	105 (14.3)	4 (17.4)	42 (16.2)	102 (16.5)	
University/college	867 (53.1)	398 (54.3)	15 (65.2)	134 (51.5)	320 (51.9)	
Graduate degree	146 (8.9)	61 (8.3)	1 (4.3)	19 (7.3)	65 (10.5)	
Marital status						0.96
Married/cohabiting	1,516 (92.8)	680 (92.8)	22 (95.7)	241 (92.7)	573 (92.9)	
Single/divorced/widowed	117 (7.2)	53 (7.2)	1 (4.3)	19 (7.3)	44 (7.1)	
Residence						<0.001
Urban	1,094 (67.0)	460 (62.8)	10 (43.5)	182 (70.0)	442 (71.6)	
Peri-urban/suburban	301 (18.4)	145 (19.8)	4 (17.4)	41 (15.8)	111 (18.0)	
Rural	238 (14.6)	128 (17.5)	9 (39.1)	37 (14.2)	64 (10.4)	
Household income quintile	3.0 (2.0–4.0)	3.0 (2.0–5.0)	4.0 (2.5–4.0)	4.0 (2.0–5.0)	3.0 (2.0–4.0)	0.45
Pre-pregnancy BMI	21.9 (19.9–23.7)	21.8 (19.9–23.8)	22.4 (20.1–24.1)	21.5 (19.9–23.3)	22.1 (20.0–23.8)	0.43
Primiparity	1,176 (72.0)	541 (73.8)	19 (82.6)	175 (67.3)	441 (71.5)	0.15
Pregnancy and delivery characteristics						
Gestational diabetes	135 (8.3)	60 (8.2)	2 (8.7)	29 (11.2)	44 (7.1)	0.27
Hypertensive disorders	82 (5.0)	44 (6.0)	1 (4.3)	12 (4.6)	25 (4.1)	0.42
Gestational age, week	39.1 (38.1–40.1)	39.2 (38.1–40.1)	39.0 (38.0–39.8)	39.1 (38.0–40.1)	39.0 (38.0–40.1)	0.66
Birth weight, g	3,283 (2,945–3,622)	3,284 (2,961–3,648)	3,194 (2,837–3,471)	3,319 (2,946–3,654)	3,250 (2,933–3,576)	0.27
Birth length, cm	50.0 (48.6–51.3)	49.9 (48.5–51.1)	49.9 (48.6–50.9)	49.9 (48.6–51.6)	50.1 (48.7–51.4)	0.43
Mode of delivery						0.1
Vaginal delivery	881 (53.9)	400 (54.6)	17 (73.9)	128 (49.2)	336 (54.5)	
Cesarean delivery	752 (46.1)	333 (45.4)	6 (26.1)	132 (50.8)	281 (45.5)	
Neonatal complications	127 (7.8)	54 (7.4)	2 (8.7)	16 (6.2)	55 (8.9)	0.52
Child characteristics at survey						
Sex						0.7
Male	833 (51.0)	364 (49.7)	12 (52.2)	131 (50.4)	326 (52.8)	
Female	800 (49.0)	369 (50.3)	11 (47.8)	129 (49.6)	291 (47.2)	
Age at survey, months	53.4 (44.3–62.7)	53.9 (43.9–63.1)	53.2 (44.3–62.1)	54.2 (43.9–63.5)	53.1 (44.7–62.1)	0.82
Current weight status						0.002
Underweight	65 (4.0)	26 (3.5)	1 (4.3)	11 (4.2)	27 (4.4)	
Normal weight	1,110 (68.0)	522 (71.2)	12 (52.2)	149 (57.3)	427 (69.2)	
Overweight	290 (17.8)	124 (16.9)	8 (34.8)	61 (23.5)	97 (15.7)	
Obese	168 (10.3)	61 (8.3)	2 (8.7)	39 (15.0)	66 (10.7)	

Data are no. (%) or median (IQR). p values were calculated using Chi-square or Fisher’s exact tests for categorical variables and Kruskal–Wallis tests for continuous variables. All continuous variables departed significantly from normality on Shapiro–Wilk testing (*p* < 0.05); non-parametric tests were therefore used exclusively.

### 3.2 Breastfeeding patterns by return-to-work timing

**Figure 1:**
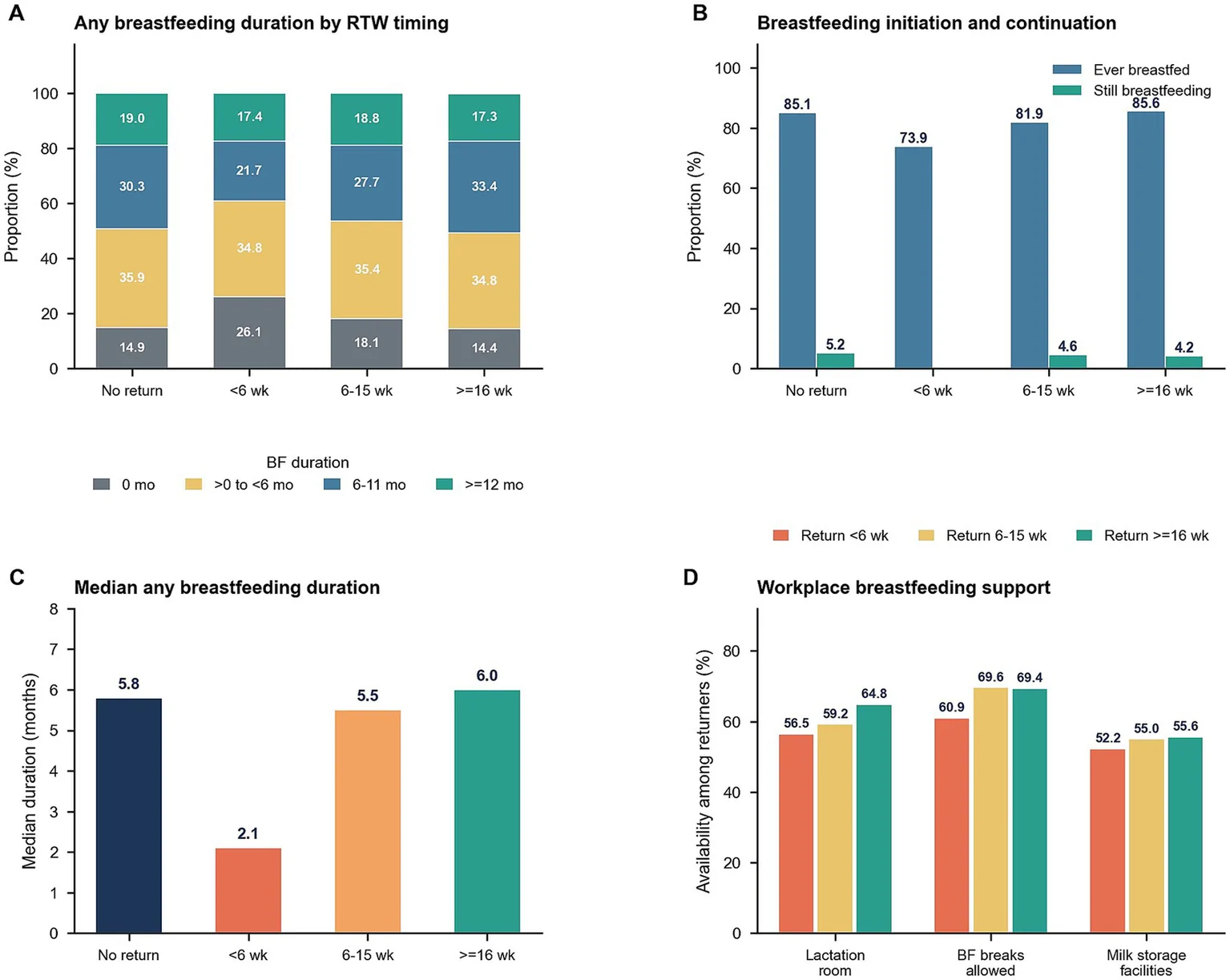
Breastfeeding patterns and workplace support by return-to-work timing. Breastfeeding duration, initiation, continuation, and workplace lactation-support indicators are shown by maternal return-to-work timing. **(A)** Harmonized any-breastfeeding duration categories. **(B)** Ever breastfeeding and continued breastfeeding at survey. **(C)** Median any breastfeeding duration. **(D)** Lactation-room access, breastfeeding breaks, and milk-storage availability among mothers who returned to paid work. BF, breastfeeding; RTW, return to work.

Overall, 1,382 mothers (84.6%) had ever breastfed (Table 2, Figure 1B). Breastfeeding initiation rates were 85.1% among non-returners, 73.9% among mothers returning before 6 weeks, 81.9% among 6–15-week returners, and 85.6% among ≥16-week returners; however, differences across groups did not reach statistical significance (*p* = 0.26). None of the mothers in the <6-week return group were still breastfeeding at the time of the survey (0.0%), compared with 5.2% of non-returners, 4.6% of 6–15-week returners, and 4.2% of ≥16-week returners (overall *p* = 0.60).

**Table 2:** Breastfeeding initiation and duration patterns by maternal return-to-work timing.

Breastfeeding characteristic	Overall (*N* = 1,633)	No return (*n* = 733)	Return <6 wk (*n* = 23)	Return 6–15 wk (*n* = 260)	Return ≥16 wk (*n* = 617)	*p*-value
Breastfeeding initiation						
Ever breastfed	1,382 (84.6)	624 (85.1)	17 (73.9)	213 (81.9)	528 (85.6)	0.26
Time to first breastfeed						0.37
Within 1 h	797 (57.7)	347 (55.6)	13 (76.5)	128 (60.1)	309 (58.5)	
1–24 h	514 (37.2)	239 (38.3)	3 (17.6)	78 (36.6)	194 (36.7)	
>24 h	71 (5.1)	38 (6.1)	1 (5.9)	7 (3.3)	25 (4.7)	
Exclusive breastfeeding duration						
Duration categories						0.42
Never	460 (28.2)	204 (27.8)	8 (34.8)	76 (29.2)	172 (27.9)	
<3 mo	639 (39.1)	281 (38.3)	12 (52.2)	102 (39.2)	244 (39.5)	
3–5 mo	349 (21.4)	156 (21.3)	1 (4.3)	50 (19.2)	142 (23.0)	
≥6 mo	185 (11.3)	92 (12.6)	2 (8.7)	32 (12.3)	59 (9.6)	
Duration, months	1.3 (0.0–4.0)	1.2 (0.0–4.5)	0.6 (0.0–2.1)	1.2 (0.0–3.6)	1.4 (0.0–3.8)	0.51
Any breastfeeding duration						
Duration categories						0.03
Never breastfed (0 mo)	251 (15.4)	109 (14.9)	6 (26.1)	47 (18.1)	89 (14.4)	
<6 mo	578 (35.4)	263 (35.9)	8 (34.8)	92 (35.4)	215 (34.8)	
6–11 mo	505 (30.9)	222 (30.3)	5 (21.7)	72 (27.7)	206 (33.4)	
≥12 mo	299 (18.3)	139 (19.0)	4 (17.4)	49 (18.8)	107 (17.3)	
Duration, months	5.8 (1.3–10.0)	5.8 (1.3–9.9)	2.1 (0.0–7.8)	5.5 (1.2–10.6)	6.0 (1.6–9.8)	0.41
Still breastfeeding at survey	76 (4.7)	38 (5.2)	0 (0.0)	12 (4.6)	26 (4.2)	0.6
Complementary feeding						
Formula introduction before 6 mo	705 (43.2)	310 (42.3)	7 (30.4)	120 (46.2)	268 (43.4)	0.44
Age at formula introduction						0.83
<1 mo	444 (63.0)	196 (63.2)	6 (85.7)	79 (65.8)	163 (60.8)	
1-2 mo	125 (17.7)	59 (19.0)	1 (14.3)	15 (12.5)	50 (18.7)	
3-4 mo	92 (13.0)	38 (12.3)	0 (0.0)	18 (15.0)	36 (13.4)	
5-6 mo	43 (6.1)	16 (5.2)	0 (0.0)	8 (6.7)	19 (7.1)	
Timing inconsistent/unknown	1 (0.1)	1 (0.3)	0 (0.0)	0 (0.0)	0 (0.0)	
Age at solid food introduction						0.37
<4 mo	173 (10.6)	83 (11.3)	2 (8.7)	20 (7.7)	68 (11.0)	
4-5 mo	591 (36.2)	247 (33.7)	8 (34.8)	98 (37.7)	238 (38.6)	
≥6 mo	869 (53.2)	403 (55.0)	13 (56.5)	142 (54.6)	311 (50.4)	

Data are no. (%) or median (IQR). Percentages for time to first breastfeed are among children ever breastfed; percentages for age at formula introduction are among children receiving formula before 6 months. Any-breastfeeding duration was harmonized with breastfeeding initiation before analysis: mothers reporting no initiation were classified as never breastfed and assigned a duration of 0 months (the “Never breastfed (0 mo)” category), and mothers still breastfeeding at survey were classified as ≥12 months.

In any-breastfeeding duration the median was shorter among mothers returning before 6 weeks (2.1 months, IQR 0.0–7.8) than among non-returners (5.8 months, IQR 1.3–9.9), 6–15-week returners (5.5 months, IQR 1.2–10.6), and ≥16-week returners (6.0 months, IQR 1.6–9.8); however, the overall difference in median duration did not reach statistical significance (*p* = 0.41; Figure 1C). The categorical distribution of harmonized any-breastfeeding duration differed significantly across return-to-work groups (*p* = 0.03; Table 2), driven by a higher proportion of mothers who never breastfed among those returning before 6 weeks (26.1%) compared with non-returners (14.9%), 6–15-week returners (18.1%), and ≥16-week returners (14.4%), and by more frequent 6–11-month breastfeeding among ≥16-week returners (33.4%) than in earlier return groups. Exclusive breastfeeding duration also tended to be shorter in the earliest return group (median 0.6 months for <6-week returners vs. 1.2–1.4 months across other groups; *p* = 0.51). The proportion of mothers who never breastfed was 14.9% among non-returners, 26.1% among mothers returning before 6 weeks, 18.1% among those returning at 6–15 weeks, and 14.4% among those returning at ≥16 weeks (Table 2, Figure 1A).

### 3.3 Maternal employment conditions and workplace breastfeeding support

Of the 900 mothers who returned to paid work, 753 (83.7%) received any maternity leave, with a median duration of 15.6 weeks (IQR 13.9–18.0) overall (Table 3). Maternity leave differed sharply by return timing (*p* < 0.001); no mother returning before 6 weeks received maternity leave (median 0.0 weeks), compared with a median of 13.9 weeks (IQR 0.0–16.9) for 6–15-week returners and 16.2 weeks (IQR 14.4–18.2) for ≥16-week returners. Workplace breastfeeding support was prevalent but not universal (Table 3, Figure 1D): 63.0% of employed mothers reported access to a lactation room, 69.2% had breastfeeding breaks permitted, and 55.3% had milk storage facilities. Support availability tended to be lowest among the earliest returners: lactation room access was 56.5, 59.2, and 64.8% for <6-week, 6–15-week, and ≥16-week groups, respectively, although no difference was statistically significant (*p* = 0.24, *p* = 0.68, and *p* = 0.94, respectively). Perceived negative job impact on breastfeeding was highest among mothers returning before 6 weeks (60.9%) and declined with later return (51.2% for 6–15 weeks, 43.8% for ≥16 weeks; *p* = 0.05; Table 3).

**Table 3:** Maternal employment conditions and workplace breastfeeding support by return-to-work timing.

Employment characteristic	Overall	No return to paid work	Return <6 wk	Return 6–15 wk	Return ≥16 wk	*p*-value
Employment conditions						
Employed before pregnancy	1,172 (71.8)	272 (37.1)	23 (100.0)	260 (100.0)	617 (100.0)	<0.001
Any maternity leave	753 (83.7)	—	0 (0.0)	155 (59.6)	598 (96.9)	<0.001
Maternity leave duration, wk	15.6 (13.9–18.0)	—	0.0 (0.0–0.0)	13.9 (0.0–16.9)	16.2 (14.4–18.2)	<0.001
Workplace support						
Lactation room available	567 (63.0)	—	13 (56.5)	154 (59.2)	400 (64.8)	0.24
Breastfeeding breaks allowed	623 (69.2)	—	14 (60.9)	181 (69.6)	428 (69.4)	0.68
Milk storage facilities	498 (55.3)	—	12 (52.2)	143 (55.0)	343 (55.6)	0.94
Healthcare provider discussion and perceptions						
HP discussed breastfeeding and work	456 (50.7)	—	12 (52.2)	137 (52.7)	307 (49.8)	0.72
Job negatively influenced BF duration	417 (46.3)	—	14 (60.9)	133 (51.2)	270 (43.8)	0.05
Workplace made breastfeeding easy	433 (48.1)	—	10 (43.5)	129 (49.6)	294 (47.6)	0.78

Data are no. (%) or median (IQR). Except for employed before pregnancy, overall estimates and *p*-values for return-related employment conditions are among 900 mothers who returned to paid work. HP, health care provider; BF, breastfeeding.

### 3.4 Return-to-work timing and child overweight/obesity

**Figure 2:**
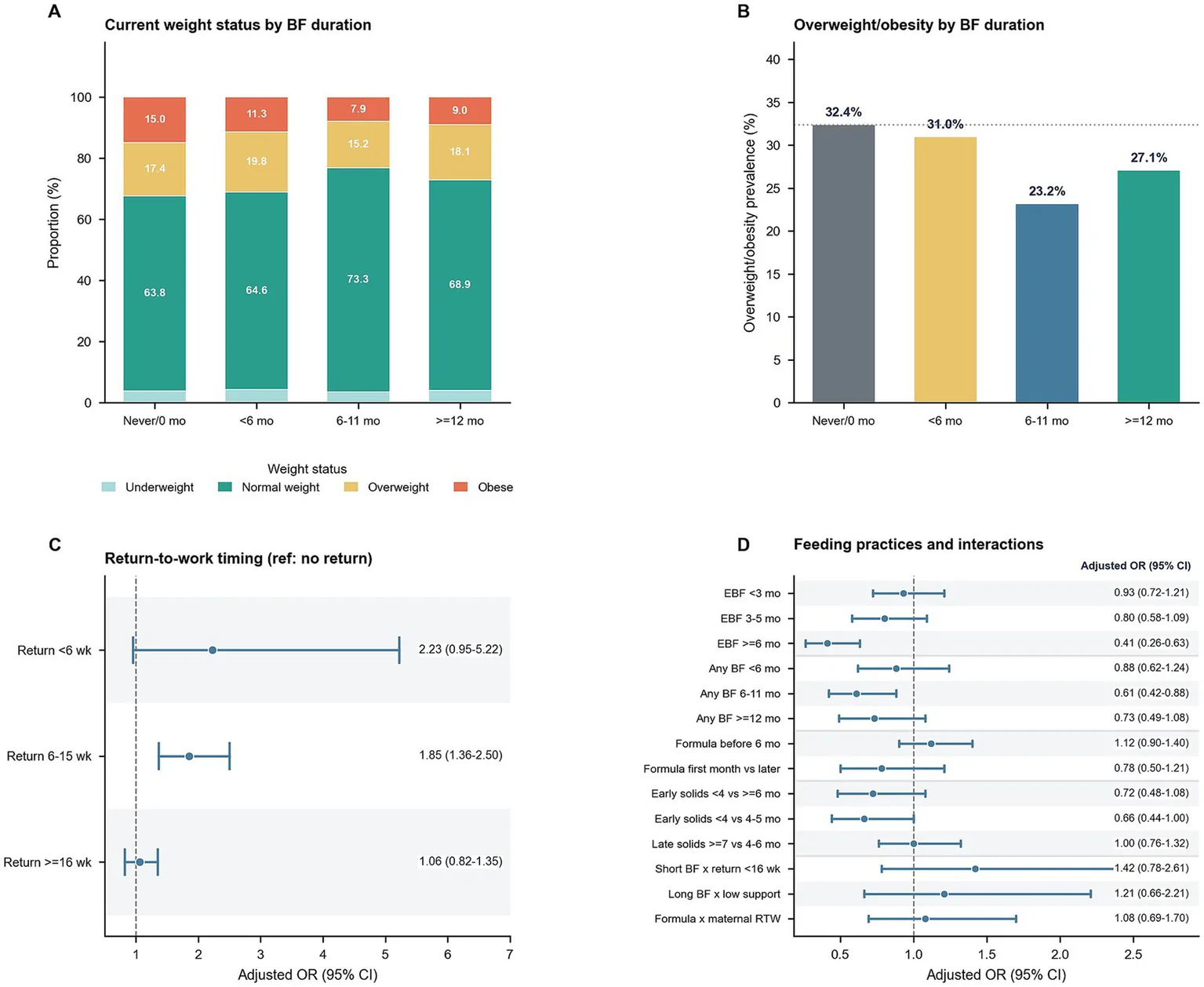
Child weight status and overweight/obesity associations. Child weight status and adjusted associations with overweight/obesity are shown by breastfeeding and return-to-work exposures. **(A)** Weight-status categories by breastfeeding duration. **(B)** Combined overweight/obesity prevalence by breastfeeding duration. **(C)** Adjusted ORs by return-to-work timing. **(D)** Adjusted ORs for feeding practices and feeding–work interaction terms. OR, odds ratio; CI, confidence interval; BF, breastfeeding.

In multivariable logistic regression adjusted for maternal age, education, household income, residence, pre-pregnancy BMI, parity, gestational age, birth weight, any breastfeeding duration, formula introduction before 6 months, child sex, and child age at survey, return-to-work timing was significantly associated with child overweight/obesity for the intermediate-return category (Table 4, Figure 2C). Relative to children of non-returning mothers, adjusted ORs were 1.85 (95% CI 1.36–2.50) for return at 6–15 weeks and 1.06 (95% CI 0.82–1.35) for return at ≥16 weeks (Table 4). The estimate for return before 6 weeks (OR 2.23, 95% CI 0.95–5.22) rests on 23 mothers and 10 events; its interval is compatible with both a null and a large association, and it is reported solely for completeness of the pre-specified categorization. No inference should be drawn from this subgroup. The test for linear trend across ordered return-to-work categories was not significant (*p* = 0.30), consistent with a non-monotonic pattern in which the 6–15-week group showed the strongest association. At the same time, ≥16-week returners did not differ meaningfully from non-returners. After Benjamini–Hochberg FDR correction across primary and secondary regression models within the overweight/obesity outcome family, the following associations retained significance at *q* < 0.05: return at 6–15 weeks (adjusted OR 1.85), exclusive breastfeeding ≥6 months (adjusted OR 0.41), and any breastfeeding 6–11 months (adjusted OR 0.61). The workplace support-stratified interaction (*p* < 0.001) was pre-designated as an exploratory analysis and was not subject to FDR correction.

**Table 4:** Multivariable logistic regression models of child overweight/obesity on maternal return-to-work timing.

Variable	Crude OR (95% CI)	Adjusted OR (95% CI)
Return-to-work timing		
No return to paid work (reference)	1 [reference]	1 [reference]
Return <6 wk	2.28 (0.98–5.28)	2.23 (0.95–5.22)
Return 6–15 wk	1.85 (1.37–2.50)	1.85 (1.36–2.50)
Return ≥16 wk	1.06 (0.83–1.36)	1.06 (0.82–1.35)
Covariates		
Maternal age, per year		0.98 (0.96–1.01)
Child sex, male vs. female		1.41 (1.13–1.75)
Child age at survey, per mo		1.01 (1.00–1.02)
Pre-pregnancy BMI, per unit		0.99 (0.96–1.02)
Any breastfeeding duration, per mo		0.99 (0.98–1.00)
Formula before 6 mo, yes vs. no		1.12 (0.90–1.40)
Urban residence, yes vs. no		0.91 (0.72–1.15)
Higher maternal education, yes vs. no		0.99 (0.79–1.24)

Adjusted model included maternal age, education, household income, residence, pre-pregnancy BMI, parity, gestational age, birth weight, breastfeeding duration, formula introduction, child sex, and age at survey. OR, odds ratio; CI, confidence interval. Test for trend across ordered return-to-work categories: *p* = 0.30.

### 3.5 Child anthropometric indicators by breastfeeding duration

The individual weight-status categories (underweight, normal weight, overweight, and obese) are presented in Table 5 and Figure 2A; the overall between-group difference across the four categories did not reach statistical significance (*p* = 0.08). The combined overweight/obesity prevalence differed significantly across breastfeeding groups (*p* = 0.01): 32.4% for never/0 months, 31.0% for <6 months, 23.2% for 6–11 months, and 27.1% for ≥12 months (Figure 2B), with children breastfed for 6–11 months showing the lowest overweight/obesity prevalence. The full weight-status distribution (underweight, normal weight, overweight, and obese) is shown in Figure 2A; between-group differences across the four weight categories did not reach statistical significance (*p* = 0.08). Rapid weight gain from birth to 24 months was recorded in 27.5–35.2% of children across breastfeeding groups (*p* = 0.21), and growth faltering in 30.9–37.3% (*p* = 0.40).

**Table 5:** Child anthropometric indicators and growth-record measures by breastfeeding duration.

Anthropometric measure	Never breastfed	<6 months	6–11 months	≥12 months	*p*-value
Current anthropometrics					
Age at survey, mo	52.3 (10.7)	53.8 (10.0)	53.7 (10.9)	53.7 (10.3)	0.32
Weight, kg	17.8 (3.2)	17.9 (2.8)	17.6 (2.9)	17.9 (2.7)	0.42
Height, cm	105.2 (7.1)	105.9 (6.7)	105.8 (7.3)	106.2 (6.9)	0.47
BMI	16.0 (2.5)	16.0 (2.3)	15.8 (2.3)	15.9 (2.3)	0.33
Age- and sex-standardized indicators					
Weight *z*-score	0.05 (1.05)	0.02 (1.01)	−0.08 (0.98)	0.06 (0.96)	0.17
Height *z*-score	−0.01 (0.96)	−0.05 (0.99)	−0.00 (1.02)	0.12 (1.00)	0.13
BMI *z*-score	0.05 (1.05)	0.05 (1.00)	−0.07 (0.99)	−0.02 (0.98)	0.23
Weight status categories					0.08
Underweight	8 (3.9)	27 (4.3)	18 (3.6)	12 (4.0)	
Normal weight	132 (63.8)	402 (64.6)	370 (73.3)	206 (68.9)	
Overweight	36 (17.4)	123 (19.8)	77 (15.2)	54 (18.1)	
Obese	31 (15.0)	70 (11.3)	40 (7.9)	27 (9.0)	
Overweight/obese combined	67 (32.4)	193 (31.0)	117 (23.2)	81 (27.1)	0.01
Historical growth-record indicators					
Rapid weight gain, birth to 24 mo	57 (27.5)	205 (33.0)	178 (35.2)	92 (30.8)	0.21
Growth faltering, length/height	64 (30.9)	232 (37.3)	183 (36.2)	111 (37.1)	0.4

Data are mean (SD) or no. (%). *Z*-scores were computed using WHO 2006 Child Growth Standards (WHO Anthro version 3.2.2 for children aged <5 years; AnthroPlus version 1.0.4 for children aged ≥5 years), standardized by age and sex. Means (SD) are presented for interpretability; all reported *p*-values for continuous measures were derived from Kruskal–Wallis tests, as these variables departed significantly from normality on Shapiro–Wilk testing. Only current anthropometry and source-supported historical growth-record indicators are reported.

### 3.6 Feeding practices and child overweight/obesity

Associations between specific feeding practices and childhood overweight/obesity are shown in Table 6 and Figure 2D. For exclusive breastfeeding, a graded inverse pattern was observed, with statistically significant evidence confined to the ≥6-month category: adjusted ORs were 0.93 (95% CI 0.72–1.21) for <3 months, 0.80 (95% CI 0.58–1.09) for 3–5 months, and 0.41 (95% CI 0.26–0.63) for ≥6 months, all compared with never exclusively breastfed. For any breastfeeding duration, 6–11 months was inversely associated with overweight/obesity (adjusted OR 0.61, 95% CI 0.42–0.88), while <6 months (adjusted OR 0.88, 95% CI 0.62–1.24) and ≥12 months (adjusted OR 0.73, 95% CI 0.49–1.08) did not reach significance. Formula introduction before 6 months was not significantly associated with overweight/obesity (adjusted OR 1.12, 95% CI 0.90–1.40), nor was formula introduction in the first month versus later (adjusted OR 0.78, 95% CI 0.50–1.21). Complementary feeding timing showed no clear independent association with overweight/obesity. However, early solids <4 months versus 4–5 months had a borderline inverse estimate: early solids <4 versus ≥6 months (adjusted OR 0.72, 95% CI 0.48–1.08), <4 versus 4-5 months (adjusted OR 0.66, 95% CI 0.44–1.00), and late solids ≥7 versus 4–6 months (adjusted OR 1.00, 95% CI 0.76–1.32) (Table 6). Three pre-specified feeding–work interaction terms were examined. None showed clear evidence of association after multivariable adjustment: short breastfeeding (<6 months) × early return (<16 weeks): adjusted OR 1.42 (95% CI 0.78–2.61); long breastfeeding (≥6 months) × low workplace support: adjusted OR 1.21 (95% CI 0.66–2.21); formula use × maternal return to work: adjusted OR 1.08 (95% CI 0.69–1.70) (Table 6). The corresponding crude interaction terms were larger (short BF × early return: crude OR 1.98, 95% CI 1.38–2.83), suggesting attenuation after adjustment for measured covariates.

**Table 6:** Associations of breastfeeding duration and feeding practices with child overweight/obesity.

Feeding practice	Crude OR (95% CI)	Adjusted OR (95% CI)
Exclusive breastfeeding duration		
Never breastfed (reference)	1 [reference]	1 [reference]
<3 mo	0.93 (0.71–1.20)	0.93 (0.72–1.21)
3–5 mo	0.78 (0.57–1.06)	0.80 (0.58–1.09)
≥6 mo	0.42 (0.27–0.65)	0.41 (0.26–0.63)
Any breastfeeding duration		
Never breastfed (reference)	1 [reference]	1 [reference]
<6 mo	0.94 (0.67–1.32)	0.88 (0.62–1.24)
6–11 mo	0.63 (0.44–0.90)	0.61 (0.42–0.88)
≥12 mo	0.78 (0.53–1.14)	0.73 (0.49–1.08)
Formula feeding patterns		
Formula before 6 mo, yes vs. no	1.16 (0.94–1.45)	1.12 (0.90–1.40)
Formula in first month vs. later	0.80 (0.52–1.21)	0.78 (0.50–1.21)
Complementary feeding timing		
Early solids, <4 mo vs. ≥6 mo	0.70 (0.47–1.03)	0.72 (0.48–1.08)
Early solids, <4 mo vs. 4-5 mo	0.64 (0.42–0.95)	0.66 (0.44–1.00)
Late solids, ≥7 mo vs. 4–6 mo	1.01 (0.77–1.33)	1.00 (0.76–1.32)
Feeding-work interaction effects		
Short BF × return <16 wk	1.98 (1.38–2.83)	1.42 (0.78–2.61)
Long BF × low workplace support	1.80 (1.22–2.66)	1.21 (0.66–2.21)
Formula use × maternal return to work	1.32 (1.03–1.69)	1.08 (0.69–1.70)

Adjusted models included maternal age, education, household income, residence, pre-pregnancy BMI, parity, gestational age, birth weight, return-to-work timing, child sex, and age at survey unless the exposure was part of an interaction model. OR, odds ratio; CI, confidence interval; BF, breastfeeding.

### 3.7 Sensitivity analyses

**Figure 3:**
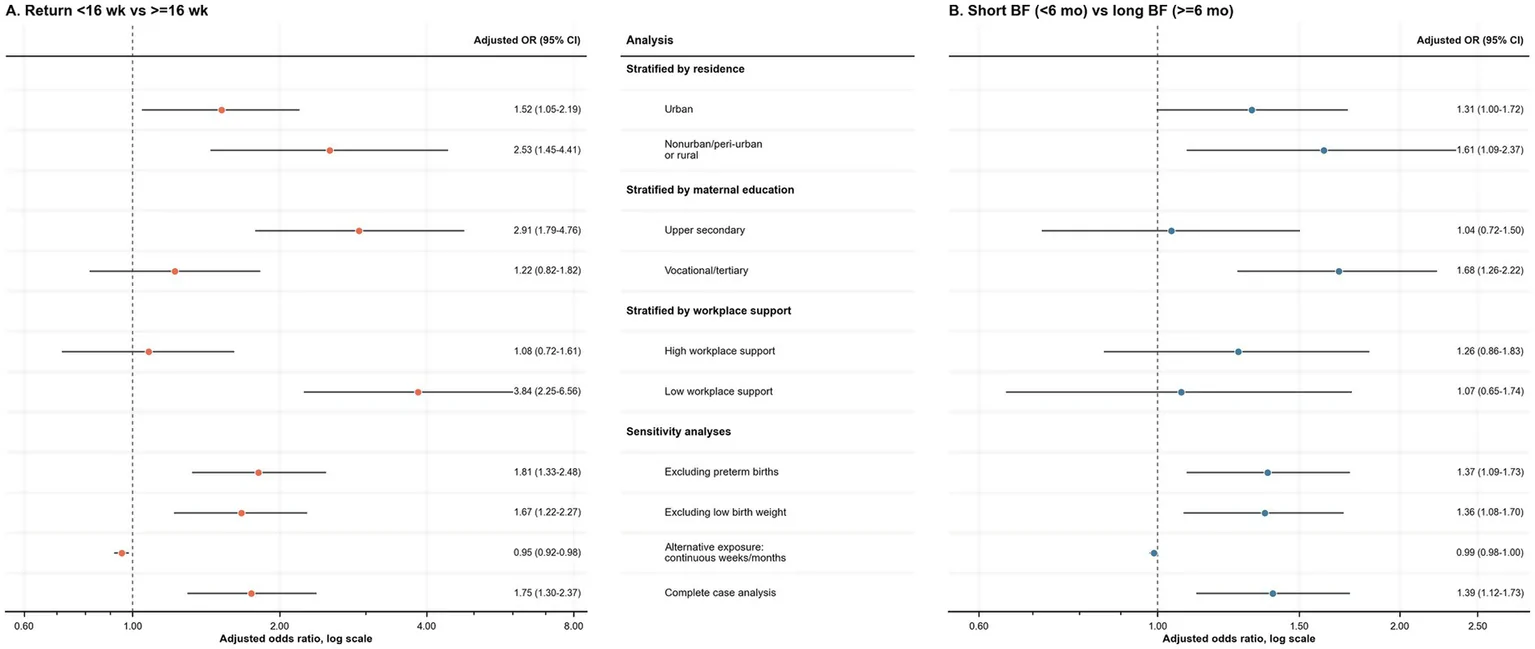
Stratified and sensitivity analyses. Adjusted ORs for child overweight/obesity are shown across stratified and sensitivity analyses. **(A)** Return before 16 weeks versus return at ≥16 weeks among mothers who resumed paid work during the first postpartum year. **(B)** Short breastfeeding duration (<6 months) versus longer breastfeeding duration (≥6 months). Stratified analyses include residence, maternal education, and workplace support; sensitivity analyses include exclusion of preterm births, exclusion of low-birth-weight infants, continuous exposure modeling, and complete-case analysis. OR, odds ratio; CI, confidence interval; BF, breastfeeding.

Stratified analyses suggested heterogeneity in the association between return-to-work timing and childhood overweight/obesity (Table 7, Figure 3). For return <16 weeks versus ≥16 weeks, adjusted ORs were 1.52 (95% CI 1.05–2.19) among urban families and 2.53 (95% CI 1.45–4.41) among non-urban, peri-urban, or rural families (*p*-for interaction = 0.16). By maternal education, the association was stronger among mothers with upper secondary education (OR 2.91, 95% CI 1.79–4.76) than among mothers with vocational or tertiary education (OR 1.22, 95% CI 0.82–1.82; *p*-for interaction = 0.009). Workplace support showed the clearest heterogeneity: return <16 weeks was not clearly associated with overweight/obesity among mothers with high workplace support (OR 1.08, 95% CI 0.72–1.61), whereas the association was markedly stronger among mothers with low workplace support (OR 3.84, 95% CI 2.25–6.56; *p*-for interaction<0.001).

**Table 7:** Stratified and sensitivity analyses for maternal return-to-work timing, breastfeeding, and child overweight/obesity.

Analysis	Return <16 wk vs. ≥16 wk OR (95% CI)	Short BF (<6 mo) vs. long BF (≥6 mo) OR (95% CI)	*p*-for interaction
Stratified by residence			
Urban	1.52 (1.05–2.19)	1.31 (1.00–1.72)	0.16
Non-urban/peri-urban or rural	2.53 (1.45–4.41)	1.61 (1.09–2.37)	
Stratified by maternal education			
Upper secondary	2.91 (1.79–4.76)	1.04 (0.72–1.50)	0.009
Vocational/tertiary	1.22 (0.82–1.82)	1.68 (1.26–2.22)	
Stratified by workplace support			
High workplace support	1.08 (0.72–1.61)	1.26 (0.86–1.83)	<0.001
Low workplace support	3.84 (2.25–6.56)	1.07 (0.65–1.74)	
Sensitivity analyses			
Excluding preterm births	1.81 (1.33–2.48)	1.37 (1.09–1.73)	
Excluding low birth weight	1.67 (1.22–2.27)	1.36 (1.08–1.70)	
Alternative exposure: per 1 week later return / per 1 additional month of breastfeeding	0.95 (0.92–0.98)	0.99 (0.98–1.00)	
Complete case analysis	1.75 (1.30–2.37)	1.39 (1.12–1.73)	

Models adjusted for maternal age, education, household income, residence, pre-pregnancy BMI, parity, gestational age, birth weight, child sex, and age at survey. The *p*-for interaction is for the return-timing exposure within each stratified analysis. BF, breastfeeding; OR, odds ratio; CI, confidence interval.

For short (<6 months) versus long (≥6 months) breastfeeding, adjusted ORs were 1.31 (95% CI 1.00–1.72) among urban families and 1.61 (95% CI 1.09–2.37) among non-urban, peri-urban, or rural families. Corresponding estimates were 1.04 (95% CI 0.72–1.50) among mothers with upper secondary education and 1.68 (95% CI 1.26–2.22) among mothers with vocational or tertiary education. Estimates by workplace support were 1.26 (95% CI 0.86–1.83) for high support and 1.07 (95% CI 0.65–1.74) for low support (Table 7, Figure 3B).

Excluding preterm births yielded an adjusted OR of 1.81 (95% CI 1.33–2.48) for return <16 versus ≥16 weeks; excluding low-birth-weight infants yielded an adjusted OR of 1.67 (95% CI 1.22–2.27). When return-to-work timing was modeled as a continuous variable, each later postpartum week of return was associated with 5% lower odds of child overweight/obesity (adjusted OR 0.95, 95% CI 0.92–0.98). For breastfeeding duration modeled continuously, each additional month was associated with approximately 1% lower odds of overweight/obesity (adjusted OR 0.99, 95% CI 0.98–1.00). Complete-case analysis in the 1,487 dyads with complete covariate data yielded an adjusted OR of 1.75 (95% CI 1.30–2.37) for the primary return-timing comparison and 1.39 (95% CI 1.12–1.73) for short versus long breastfeeding. These agree closely with the multiply imputed estimates: point estimates differ by less than 6%, all intervals overlap substantially, and no association changed in significance. No substantive difference therefore emerged between the two approaches, consistent with the low proportion of missing data and the missing-completely-at-random pattern.

## 4 Discussion

This study is cross-sectional with retrospectively reported exposures, and no temporal ordering between workplace conditions, feeding practices, and child weight status can be established from these data. Specifically, return to paid work at 6–15 postpartum weeks was associated with higher adjusted odds of child overweight/obesity compared with no return (adjusted OR 1.85, 95% CI 1.36–2.50), whereas return before 6 weeks showed an imprecise positive estimate (adjusted OR 2.23, 95% CI 0.95–5.22) and return at ≥16 weeks was not associated with excess odds (adjusted OR 1.06, 95% CI 0.82–1.35). The adjusted feeding–work interaction terms were not statistically significant, so these results cannot establish that early return combined with short breastfeeding predicts obesity. They equally cannot exclude it. The study was powered for a main effect rather than for interaction, and the intervals around the interaction terms remain wide enough to accommodate clinically meaningful departures from multiplicativity—0.78 to 2.61 for short breastfeeding × early return, 0.66–2.21 for long breastfeeding × low workplace support, and 0.69–1.70 for formula use × maternal return. A joint effect can therefore be neither demonstrated nor ruled out here, and adequately powered studies are needed to resolve the question. In contrast, stratified analyses suggested that the workplace context may modify risk: return before 16 weeks was not clearly associated with overweight/obesity among mothers with high workplace support (OR 1.08, 95% CI 0.72–1.61), whereas the association was markedly stronger among those with low workplace support (OR 3.84, 95% CI 2.25–6.56; *p*-for interaction <0.001). These findings suggest that workplace conditions may identify a policy-relevant subgroup in which earlier return to work is most strongly associated with child adiposity, although causal mediation through breastfeeding continuation cannot be inferred from these data.

Breastfeeding initiation rates did not differ significantly across return-to-work groups (*p* = 0.26), nor did median any-breastfeeding duration (*p* = 0.41), although the categorical distribution of any-breastfeeding duration did differ (*p* = 0.03); all three *p*-values refer to between-group comparisons across the four return-to-work categories. Together, this pattern suggests that the employment—a simple breastfeeding–discontinuation pathway cannot explain the obesity association. Ever breastfeeding was common across all groups, and exclusive breastfeeding duration did not differ significantly by return-to-work category (*p* = 0.42), nor did median continuous any-breastfeeding duration (*p* = 0.41). Although mothers returning before 6 weeks had descriptively shorter median breastfeeding duration, this subgroup was small (*n* = 23), and the overall between-group difference was not statistically significant. These findings are consistent with prior studies suggesting that maternal employment alone does not uniformly determine child weight outcomes, particularly when employment conditions, household resources, and infant-feeding support vary across settings (51, 52). However, the present results also differ from studies in which maternal work hours were directly associated with child BMI (53) because the current data indicate that risk is concentrated in specific timing and support strata rather than distributed linearly across all return-to-work categories.

Breastfeeding showed a stronger association with childhood overweight/obesity than return-to-work timing. Exclusive breastfeeding for ≥6 months was associated with substantially lower adjusted odds of overweight/obesity compared with never exclusive breastfeeding (adjusted OR 0.41, 95% CI 0.26–0.63). Any breastfeeding for 6–11 months was also associated with lower odds (adjusted OR 0.61, 95% CI 0.42–0.88), whereas any breastfeeding for ≥12 months was not significantly associated (adjusted OR 0.73, 95% CI 0.49–1.08). This pattern does not support a simple linear dose–response relationship across all breastfeeding-duration categories; rather, it suggests that the inverse association was most evident for sustained exclusive breastfeeding and for any breastfeeding lasting 6–11 months. Introduction of formula before 6 months was not independently linked to overweight or obesity after adjustments. Complementary feeding indicators also showed no clear independent association, although the comparison between early solids under 4 months and 4-5 months was borderline and warrants cautious interpretation. These results align broadly with meta-analytic findings suggesting that breastfeeding might lower the risk of overweight, but due to the observational design and potential residual confounding, causal conclusions cannot be drawn (10).

Several mechanisms have been proposed to link sustained breastfeeding with lower child adiposity, none of which was measured here. Breast milk contains bioactive hormones, immunologic factors, and human milk oligosaccharides, which may influence appetite regulation, gut microbiota development, inflammatory tone, and energy balance. Early formula exposure may instead alter protein intake and growth signaling, including insulin-like growth factor-1-related pathways. In this study data, however, formula introduction before 6 months showed no independent association with overweight/obesity (54, 55). Therefore, mechanistic interpretation should be cautious: the study supports an epidemiologic association between breastfeeding duration and child weight status but does not directly demonstrate metabolic programming, insulin resistance, gut microbiome alteration, or future type 2 diabetes prevention.

Workplace support impacts maternal and child health policies. Among mothers returning to paid work, lactation support was common but incomplete: 63.0% had lactation rooms, 69.2% permitted breastfeeding breaks, and 55.3% had milk storage facilities. These support indicators did not vary significantly across return-to-work categories, though negative job influence on breastfeeding was highest among mothers returning before 6 weeks and declined with later returns. Stratified analyses showed low workplace support identified the subgroup in which returning before 16 weeks was most strongly linked to child overweight/obesity. This supports policies combining postpartum leave with lactation accommodations like private spaces, scheduled breaks, safe storage, and protection from penalties. Workplace support also indicates broader job quality factors like employment formality, scheduling autonomy, income stability, and health benefits. Biological mechanisms connecting breastfeeding and lower child adiposity include effects of milk bioactive factors, appetite regulation, gut microbiota, and early growth signals (56). In other populations, oligosaccharide exposure has been associated with gut microbial composition and with inflammatory and metabolic profiles (57–59), none of these was measured in the current study. The 16-week threshold used in the stratified analyses is an analytic cut point chosen before analysis for its correspondence to policy-relevant leave durations, not a biologically derived boundary or evidence of a sensitive period. Whether the observed associations persist into later childhood cannot be determined from cross-sectional data and requires longitudinal follow-up.

Return to paid work at 6–15 weeks postpartum was associated with a higher adjusted odds of child overweight/obesity compared with no return (adjusted OR 1.85, 95% CI 1.36–2.50), whereas return before 6 weeks showed an imprecise positive estimate and return at ≥16 weeks was not associated with excess odds. The adjusted feeding–work interaction terms were not statistically significant; therefore, the results should not be interpreted as proving that early return combined with short breastfeeding independently predicts obesity. Instead, the strongest evidence of heterogeneity came from the stratified workplace-support analysis, in which return before 16 weeks was not clearly associated with overweight/obesity among mothers with high workplace support (OR 1.08, 95% CI 0.72–1.61) but was strongly associated among those with low workplace support (OR 3.84, 95% CI 2.25–6.56; *p*-for interaction <0.001). This study characterizes return-to-work timing, workplace lactation support, and breastfeeding duration together rather than separately, which is its principal contribution; the pattern is consistent with prior evidence that maternal employment–child weight associations vary by household and employment context (60, 61).

These findings should be interpreted within the context of China’s maternal leave and workplace lactation policies. Although statutory maternity leave protections exist, the study suggests that practical access to leave and lactation accommodations remains incomplete among mothers who return to paid work. Among 900 returners, 753 (83.7%) reported any maternity leave, and median leave duration was 15.6 weeks (IQR 13.9–18.0). No mother returning before 6 weeks reported maternity leave, compared with 59.6% of 6–15-week returners and 96.9% of ≥16-week returners. Workplace lactation support was also incomplete: lactation room access was reported by 63.0%, breastfeeding breaks by 69.2%, and milk storage facilities by 55.3% of returners. Support availability was numerically lowest among the earliest returners, but these between-group differences were not statistically significant and should not be interpreted as a confirmed gradient. The perceived negative job influence on breastfeeding was highest among mothers returning before 6 weeks (60.9%) and declined with later returns (51.2% for 6–15 weeks and 43.8% for ≥16 weeks; *p* = 0.05), suggesting that early return may reflect a more constrained employment environment.

The policy implications are therefore actionable but should be framed cautiously. If these associations are causal—which this design cannot establish—extending protected postpartum leave and ensuring enforceable lactation accommodations would be expected to benefit mothers who return before 16 weeks and lack workplace support. This interpretation aligns with international maternity protection standards, which emphasize adequate leave duration, paid breastfeeding breaks, and hygienic nursing facilities (62). In China, mothers are nationally entitled to 98 days of paid maternity leave and daily paid breastfeeding breaks until the child turns 1 year, with additional provincial provisions in some settings (63, 64). Dedicated private lactation rooms, protected expression breaks, safe milk storage, supervisor training, and protection from workplace penalties are feasible measures that create structural conditions for sustained breastfeeding, and workplace breastfeeding programs have generally shown positive effects on breastfeeding outcomes in systematic reviews (65, 66). However, the apparent association with workplace support should not be interpreted as the isolated biological effect of breastfeeding continuation, because workplace support may also indicate broader job quality, including formal employment, income stability, scheduling autonomy, and access to health-related benefits.

Breastfeeding findings also require precise interpretation. Exclusive breastfeeding for ≥6 months was associated with substantially lower adjusted odds of overweight/obesity (adjusted OR 0.41, 95% CI 0.26–0.63), and any breastfeeding for 6–11 months was also inversely associated (adjusted OR 0.61, 95% CI 0.42–0.88). These results are consistent with meta-analytic evidence linking breastfeeding with lower childhood overweight/obesity risk and recommendations supporting exclusive breastfeeding in early infancy (67–69). However, any breastfeeding for ≥12 months did not reach statistical significance (adjusted OR 0.73, 95% CI 0.49–1.08), and formula introduction before 6 months was not independently associated with overweight/obesity (adjusted OR 1.12, 95% CI 0.90–1.40). These results support breastfeeding promotion as part of child obesity prevention; however, they do not establish a simple dose–response relationship across all breastfeeding categories.

The stratified findings refine the interpretation of social heterogeneity. The strongest return-timing association was not observed among urban or highly educated mothers. Return before 16 weeks versus ≥16 weeks was associated with overweight/obesity among urban families (OR 1.52, 95% CI 1.05–2.19) and showed a larger point estimate among non-urban, peri-urban, or rural families (OR 2.53, 95% CI 1.45–4.41), although the residential interaction was not significant (*p* = 0.16). By education, the association was stronger among mothers with upper secondary education (OR 2.91, 95% CI 1.79–4.76) than among those with vocational or tertiary education (OR 1.22, 95% CI 0.82–1.82; *p*-for interaction = 0.009). These patterns suggest heterogeneity by socioeconomic and employment context; however, the mechanisms cannot be determined from the available data.

This study has several strengths, including a large clinic-based sample, standardized preschool anthropometric assessment, extraction of prospectively recorded early-life growth measurements, detailed assessment of return-to-work timing and workplace lactation support, and pre-specified stratified and sensitivity analyses. Several limitations qualify these findings. The design is cross-sectional, and although growth measurements were extracted from prospectively maintained records, exposures were reported at a single visit after the outcome had developed. Temporal ordering between workplace conditions, feeding practices, and child weight status therefore cannot be established, and reverse causation cannot be excluded: mothers of children with early growth concerns may have altered feeding or employment decisions in ways that generate the associations observed in this study. No estimate reported here should be read as an intervention effect.

Selection and measurement error are both plausible. Recruitment occurred at a single urban hospital in Pingxiang, Jiangxi, a less developed province compared with the coastal regions, and the sample over-represents formally employed families with routine access to maternal–child services. The resulting bias cannot be quantified because no characteristics were retained for eligible dyads who declined or were excluded. If participating mothers were more health-conscious and more often formally employed, both lactation provision and realized maternity leave would be overstated relative to the general working population, and the association between early return and overweight/obesity would most plausibly be attenuated rather than inflated. Exposures were recalled over intervals extending to 6 years. Memory anchors and child health booklets were used to improve accuracy, and external validation in comparable populations indicates good agreement between single-item breastfeeding recall and prospective records over intervals up to 5 years. Still, no test–retest assessment was undertaken in this sample. Because feeding and employment histories were reported without knowledge of the child’s weight status, misclassification is likely to be non-differential and to bias estimates toward the null. The workplace support score compounds this concern: it was purpose-built from three items mapped to WHO and ILO Convention 183 prerequisites, has undergone no content-validity testing or expert review, and could not be verified against employer audit or direct observation of facilities.

Residual confounding remains the principal threat to validity. Child dietary intake, physical activity, sedentary and screen time, sleep duration, childcare arrangement after maternal return, and household food environment were not measured, nor were paternal BMI, working hours, or involvement in daily caregiving. Several of these plausibly lie partly on the pathway between early return and child adiposity as well as confounding it, so adjustment would not necessarily move estimates in a single direction. The net direction of this bias cannot be determined.

Finally, precision constrains interpretation. Twenty-three mothers returned before 6 weeks, and that estimate supports no inference. The study was powered for the main effect of return timing and not for interaction, which conventionally requires roughly a 4-fold larger sample; the null feeding–work interaction terms therefore represent absence of evidence rather than evidence of absence. Stratified analyses were exploratory, uncorrected for multiplicity, and hypothesis-generating. Prospective multicenter cohorts with verified workplace provision and contemporaneous feeding records are needed.

## 5 Conclusion

In this single-center cross-sectional study of 1,633 mother–child dyads, return at 6–15 postpartum weeks was the only timing category robustly associated with preschool overweight/obesity; the trend test was non-significant, and the estimate for return before 6 weeks rests on too few mothers to support any conclusion. Sustained breastfeeding was associated with lower odds. Heterogeneity by workplace lactation support was the most pronounced subgroup finding. Still, it derives from an exploratory analysis in a study powered for a main effect rather than for interaction, and all three pre-specified feeding–work interaction terms were null. These results are further constrained by the retrospective reporting of exposures, the absence of data on child diet, activity, sleep, and childcare arrangements, and the absence of paternal characteristics; residual confounding of indeterminate direction should be assumed. Workplace lactation accommodation and protected postpartum employment conditions are therefore candidate policy targets rather than demonstrated ones, and prospective multicenter cohorts with contemporaneous feeding records, verified workplace provision, and longitudinal anthropometry are needed to establish whether these associations are causal and whether they persist beyond the preschool years.

## Data Availability

The raw data supporting the conclusions of this article will be made available by the authors, without undue reservation.
